# NK cell-derived exosomes improved lung injury in mouse model of *Pseudomonas aeruginosa* lung infection

**DOI:** 10.1186/s12576-020-00776-9

**Published:** 2020-10-23

**Authors:** Ruiqi Jia, Kuili Cui, Zhenkui Li, Yuan Gao, Bianfang Zhang, Zhixia Wang, Junwei Cui

**Affiliations:** 1 Respiratory Intensive Care Unit, The First Affiliated Hospital of Xinxiang Medical University, No. 88 Jiankang Road, Xinxiang, 453003 China; 2 Tuberculosis Medicine, The First Affiliated Hospital of Xinxiang Medical University, No. 88 Jiankang Road, Xinxiang, 453003 China; 3 Respiratory Medicine, The First Affiliated Hospital of Xinxiang Medical University, No. 88 Jiankang Road, Xinxiang, 453003 China

**Keywords:** *Pseudomonas aeruginosa*, Lung infection, Natural killer cell, Macrophage, Exosome

## Abstract

**Background:**

*Pseudomonas aeruginosa* (*PA*) is one of the most common bacteria that causes lung infection in hospital. The aim of our study is to explore the role and action mechanism of NK cells in lung *PA* infection.

**Methods:**

In this present study, 2.5 × 10^8^ CFU/mouse *PA* was injected into murine trachea to make lung *PA* infection mouse model. Anti-asialo GM1 was used to inhibit NK cell. The percentage of NK cells was ensured by flow cytometry, and the M1- and M2-polarized macrophages were determined by flow cytometry, qRT-PCR, and ELISA assay. Besides, H&E staining was performed to ensure the pathological changes in lung tissues. Transmission electron microscopy and western blot were carried out to identify the exosome.

**Results:**

Here, in the mouse model of PA lung infection, NK cell depletion caused M2 polarization of lung macrophage, and exacerbated *PA*-induced lung injury. Next, our data shown that M2 macrophage polarization was enhanced when the generation of NK cell-derived exosome was blocked in the co-culture system of NK cells and macrophages. Subsequently, we demonstrated that NK cells promoted M1 macrophage polarization both in *PA*-infected macrophage and the mouse model of *PA* lung infection, and attenuated lung injury through exosome.

**Conclusion:**

Overall, our data proved that NK cell may improve *PA*-induced lung injury through promoting M1 lung macrophage polarization by secreting exosome. Our results provide a new idea for the treatment of *PA* lung infection.

**Electronic supplementary material:**

The online version of this article (10.1186/s12576-020-00776-9) contains supplementary material, which is available to authorized users.

## Background

*Pseudomonas aeruginosa* (*PA*) is a Gram-negative bacterium within the hospital environment, and the major cause of nosocomial pneumonia which mainly occurs in the patients with chronic obstructive pulmonary disease and cystic fibrosis, and the immunocompromised [1, 2]. Currently, due to the limitation of antibiotic therapy caused by the increasing multidrug resistance microbe, *PA* was listed in the most critical group of drug-resistant germs by the World Health Organization [3, 4]. The molecular mechanisms of governing immune response to *PA* lung infection remains not fully unclear.

For pulmonary bacterial infection, *PA* must overcome the innate host defense responses. During the development of *PA* infection, immune cells, such as dendritic cells, neutrophils, macrophages and natural killer (NK) cells, were recruited to the site of infection to clear *PA*, while excessive inflammation enhances bacterial infection and *PA*-induced lung injury [5]. Macrophage is an important component of the immune system in human, and plays a crucial role in immune responses, tissue homeostasis and the occurrence and development of multiple diseases [6, 7]. It was demonstrated that macrophages are plastic, and can be differentiated into two polarization phenotypes, including pro-inflammatory M1 and anti-inflammatory M2 macrophage according to the tissue microenvironment. M1-polarized macrophage promotes the development of inflammation via secreting TNF-α, IL-1β and IL-6, and M2-polarized macrophage expresses high level of IL-10 [8, 9]. In recent studies, Tian et al. revealed that during acute lung infection, the regulator of type III secretion system of *PA*, ExsA, induced the degradation of CD44, a macrophage surface protein, could obstruct the phagocytosis of macrophage [10]. Moreover, it was indicated that *PA* could lead to the production of IL-6 in lung macrophage [11]. A novel therapeutic method that does not trigger further inflammatory signalling of *PA* lung infection can be hopefully found through exploring the regulatory mechanism between *PA* infection and macrophage polarization.

Another important component of human immune system, NK cell, also acts as a necessary regulator during the defence responses to bacterial pathogen infection [12]. NK cells are CD56^+^CD3^−^ lymphocytes of the innate immune system. It was indicated that NK cells can interact with other immune cells, such as dendritic cells, B cells, neutrophils and T cells, via secreting cytokines, chemokines and exosomes [13, 14]. Alexis et al. indicated that the depletion of NK cells is closely related to mice susceptibility in the mouse model of *PA* pneumonia infection [15]. Besides, NK cells were also proved to interact with macrophage to regulate immune response in infectious disease [16]. In the mouse model of nonalcoholic steatohepatitis, NK cells participated in the development of the disease through promoting macrophage to M1 polarization [17]. In this present study, our data demonstrated that NK cells improved *PA*-induced inflammation and lung injury in the mouse model of *PA* lung infection. Importantly, NK cells play their function via inducing M1 macrophage polarization by secretion of exosomes. Our data provide a new regulatory mechanism for the development of *PA* lung infection, which is related to the interaction between NK cells and macrophage.

## Materials and methods

### Animals

All animal experiments were performed on the basis of the guidelines for the Care and Use of Laboratory Animals of the National Institutes of Health, and approved by The First Affiliated Hospital of Xinxiang Medical University. Eight- to ten-week-old C57BL/6 mice (female, 17 – 22 g) were purchased from Charles River (Beijing, China). All mice were raised in a standard environment with 12/12 h of right/dark cycle and enough food and water.

### Bacterial preparation

*PA* strain PAO1 (Institute of Microbiology, Chinese Academy of Sciences, Beijing, China) is one of the most commonly used strains in laboratory. The agarose beads which wrapped with bacterium was prepared in accordance with the previous study, and suspended in aseptic PBS solution [18]. Then, the agarose bead suspension was cultured in a sheep blood agar at 37 °C for 24 h for colony counting.

### Lung infection with PA and lung tissue collection

Mice were anesthetized using 10% chloral hydrate, and then their tracheas were exposed. Next, 50 μl agarose bead suspension with a concentration of 5 × 10^8^ CFU/ml was injected into the mice through trachea. Three days after surgery, all animals were euthanized. Next, the right lung tissues of mice were removed, and then homogenized in sterile saline. After that, lung homogenate was maintained on a sheep blood agar for 24 h at 37 °C for counting of bacterial CFU.

### Animal groups

For different experimental purposes, the mice were randomly divided into several groups. To detect the efficiency of NK cells removal, the mice of *PA* lung infection were divided into two groups: *PA* + isotype, and *PA* + NK(-). In *PA* + NK(-) group, the mice were injected with 70 μl anti-asialo GM1 through tail vein following *PA* infection. In PA + isotype group, the mice were injected with 70 μl control anti-IgG following *PA* infection. To explore the role of NK cells in lung injury induced by *PA*, the mice were divided into four groups: Control, *PA*, *PA* + isotype, and *PA* + NK(-). In control group, the mice were injected with aseptic agarose beads via trachea. In *PA* group, the mice were injected with agarose beads wrapped with *PA*. Besides, to explore the role of exosomes derived from NK cells in lung injury induced by *PA*, the NK cell-depleted mice were divided into three groups: NK(-), NK(-) + NK, and NK(-) + NK-Exo. In NK(-) group, the mice were treated with anti-asialo GM1 following *PA*. In NK(-) + NK group, the mice were co-injected with anti-asialo GM1 and NK cells via tail vein following *PA*. In NK(-) + NK-Exo group, the mice were injected with both anti-asialo GM1 and NK cell-derived exosomes through tail vein following *PA*.

### Cell culture and treatment

Mouse NK cells were from the frozen primary NK cells in our laboratory. Mouse macrophage RAW264.7 were obtained from American Type Culture Collection (ATCC, Rockville, MD USA). The NK cells were cultured in DMEM supplemented with 20% fetal bovine serum (FBS, Gibco), and RAW264.7 was maintained in DMEM supplemented with 10% FBS. All cells were cultured in an incubator with 5% CO_2_ at 37 °C.

A total of 1 × 10^5^/well NK cells were planted in the upper chamber of the transwell system (3 μm, Corning-Costar, USA), and 2 × 10^5^/well macrophages were cultured in the lower chamber. NK cells were treated with 10 μM GW4869 to block the generation of exosomes, and the cells in control group were treated with equal volume of PBS. Meantime, macrophages were infected with *PA* (MOI = 10). Then, 24 h later, the next experiments were done.

### Preparation of NK cells

NK cells were isolated using a mouse NK cell isolation kit (Miltenyi Biotec Auburn, USA). The spleen of mouse was removed and then cut into small fragments. Next, these fragments were incubated with collagenase IV for 20 min at room temperature, and then washed with 0.01 M PBS solution. After the digested tissue was resuspended in 5 ml of Histopaque 1077 (Sigma-Aldrich, St. Louis, MO, USA), another 5 ml of Histopaque 1077 was added in the upper layer, and 1 ml of FBS was added. Subsequently, the cells were centrifuged at 3000 rpm for 10 min, and splenocytes were obtained. These cells were incubated with anti-mouse CD3-FITC (eBioscience, Thermo Fisher Scientific, USA) and CD49b-PE, and then analyzed by flow cytometry.

### NK cell-derived exosomes isolation and identification

Cell culture medium was collected and then centrifuged for 15 min at 30,000 g to remove cells and cellular debris. Next, exosomes were extracted from cell culture supernatants of NK cells using ExoQuick Exosome Precipitation Solution (SBI System Biosciences, USA) in accordance with the protocol and filtered through a 200-nm filter. After that, the shape of exosomes was examined by transmission electron microscopy (HT7700, Hitachi High-technologies). Besides, RIPA lysis buffer was used to isolate the total protein from NK cells or exosomes, and 20 μg of protein from each group were separated by 12% SDS-PAGE. The surface markers of exosome, including CD63 and CD9, were detected by western blotting assay against CD63 and CD9 antibodies (Abcam, Cambridge, MA). PBS or 10 μg exosomes were incubated with macrophage following *PA* infection, and after 24 h, the next experiments were performed.

### Flow cytometry assay

Flow cytometry was carried out to detect the percentage of NK cells, macrophage, neutrophil, lymphocyte, and M1- and M2-polarized macrophage in lung tissues of mice. All tissues were cut into 1 × 1 mm^2^ pieces, and then digested with 1 mg/ml collagenase I (Roche, Indianapolis, IN) in DMEM (Gibco, USA) to obtain the single cell suspensions for flow cytometry assay. Next step, 200 μl cell suspensions were incubated with FITC-labeled CD3, FITC-labeled DX5, PE-labeled F4/80, FITC-labeled CD86, FITC-labeled F4/80 or PE-labeled CD206 antibodies (BD Biosciences, San Jose, CA, USA) for 30 min at 4 °C in the dark. Subsequently, the cells were washed for twice using PBS solution, and fixed with 1% paraformaldehyde. At last, the percentages of NK cell, and M1- and M2-polarized macrophage were analyzed using a flow cytometry (FACS Calibur, BD Biosciences).

### QRT-PCR assay

Total RNA was isolated from lung tissues or cells using TRIzol reagent (Invitrogen, Carlsbad, CA, USA). Then, cDNA was synthesized following the total RNA was reverse transcripted using an iScript cDNA Synthesis Kit (Bio-Rad Laboratories, Hercules, CA, USA). After that, the quantitative real-time PCR was carried out to detect the relative expression of RNA using SYBR green method on an ABI 7500 real-time PCR system (Applied Biosystems, USA). Finally, the relative expression of *iNOS*, *Arg-1*, TNF-α, IL-1β and IL-10 mRNAs was calculated as the method of 2^−ΔΔCt^, and normalized to U6 snRNA. The primer sequences used are as follows: *iNOS*: 5′-GACCAGATAAGGGCAAGCAC-3′ (sense) and 5′-CTTGTCTTT GACCCAGTAGC-3′ (antisense); *Arg-1*: 5′-TGCAGTGGCAGAAATCAAGA-3′ (sense) and 5′-AGCATCCACCCAAATGACA-3′ (antisense); TNF-α: 5′-ACCCTCA CACTCAGATCATCTTC-3′ (sense) and 5′-TGGTGGTTTGCTACGACGT-3′ (antisense); IL-1β: 5′-TTGTTGCTGTGGAGAAGCTGT-3′ (sense) and 5′-AACGTC ACACACCAGCAGGTT-3′ (antisense); IL-10: 5′-ATCTTAGCTAACGGAAACAAC TCCT-3′ (sense) and 5′-TAGAATGGGAACTGAGGTATCAGAG-3′ (antisense).

### ELISA assay

The concentrations of TNF-α, IL-1β and IL-10 in lung tissues, and IFN-γ in cell culture supernatant were measured by ELISA (Tianjin Anoric Biotechnology, Tianjin, China) according to the manufacture’s introduction of kits. A total of 50 μl/well standard reagents and samples were added into a 96-well plate, and then incubated for 20 h at 4 °C. Next, the samples were incubated with biotinylated antibodies for 1 h at 37 °C followed by streptavidin–HRP (100 μl per well). After that, cells were incubated with TMB solution (100 μl per well) at 37 °C and subsequently the reaction was stopped by stop solution. The absorbance value was measured by Multiskan™ FC (Thermo Fisher Scientific) at 450 nm [19].

### H&E staining

H&E staining was carried out to confirm the pathological changes in the lung tissues. Paraffin-embedded lung tissues were cut into 4-μm pieces, and then these slices were treated with dewaxed and hydrated. Then, the staining was performed according to the protocol of H&E staining kit (Solarbio, Beijing, China). At last, the sections were analyzed under a light microscope. Histological scoring of lung tissues was calculated according to previous studies [20, 21].

### Statistical analysis

All data were analyzed using SPSS 20.0 software (IBM Corporation, USA), and shown as mean ± SD. All experiments were independently repeated three times unless specified. Student’s *t*-test was performed to detect the significant difference between two groups, and one-way ANOVA analysis followed by Tukey's post hoc test were used for the comparison among three or more groups. *P* values lower than 0.05 were considered as significant.

## Results

### M2 macrophage polarization was promoted in the mouse model of PA lung infection after NK cell was eliminated

**Fig. 1:**
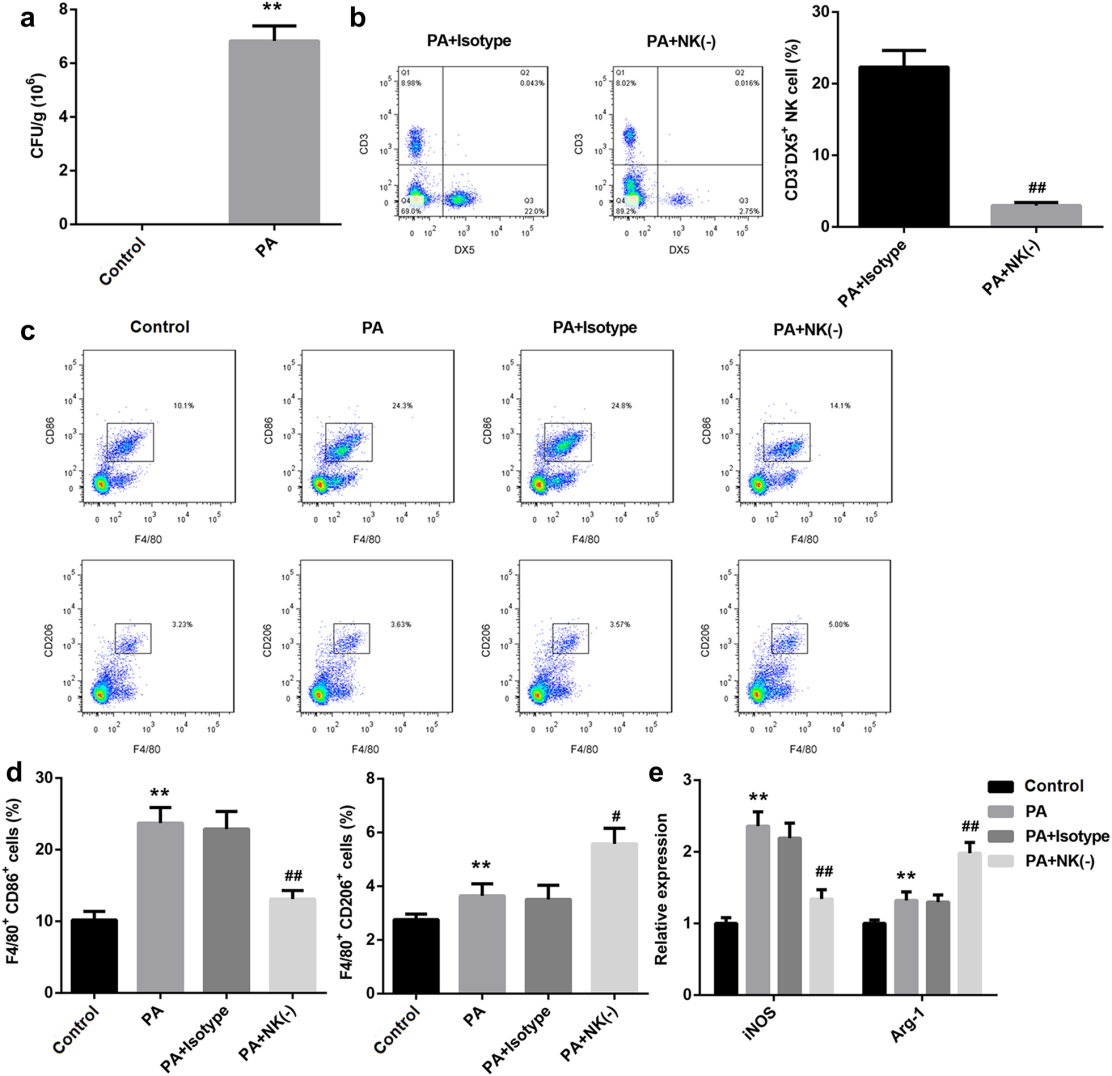
NK cells elimination promoted M2 macrophage polarization in the mouse model of *PA* lung infection. **a** The bacterial burden in the right lung tissues of mice was analyzed using CFU counting. **P < 0.01 compared with Control group. **b** Flow cytometry was carried out to analyze the number of NK cells in lung tissues of mice. NK(-): NK cell-depleted mice, ^##^*P* < 0.01 compared with *PA* + isotype group. **c**, **d** The cell numbers of M1-polarized and M2-polarized macrophages in lung tissues were ensured using flow cytometry, and then counted. **P < 0.01 compared with Control group, both ^#^*P* < 0.05 and ^##^*P* < 0.01 contrasted with *PA* group. **e** The expression of iNOS and *Arg-1* in lung tissues was measured by qRT-PCR. **P < 0.01 compared with Control group, ^##^*P* < 0.01 contrasted with *PA* group

**Fig. 2:**
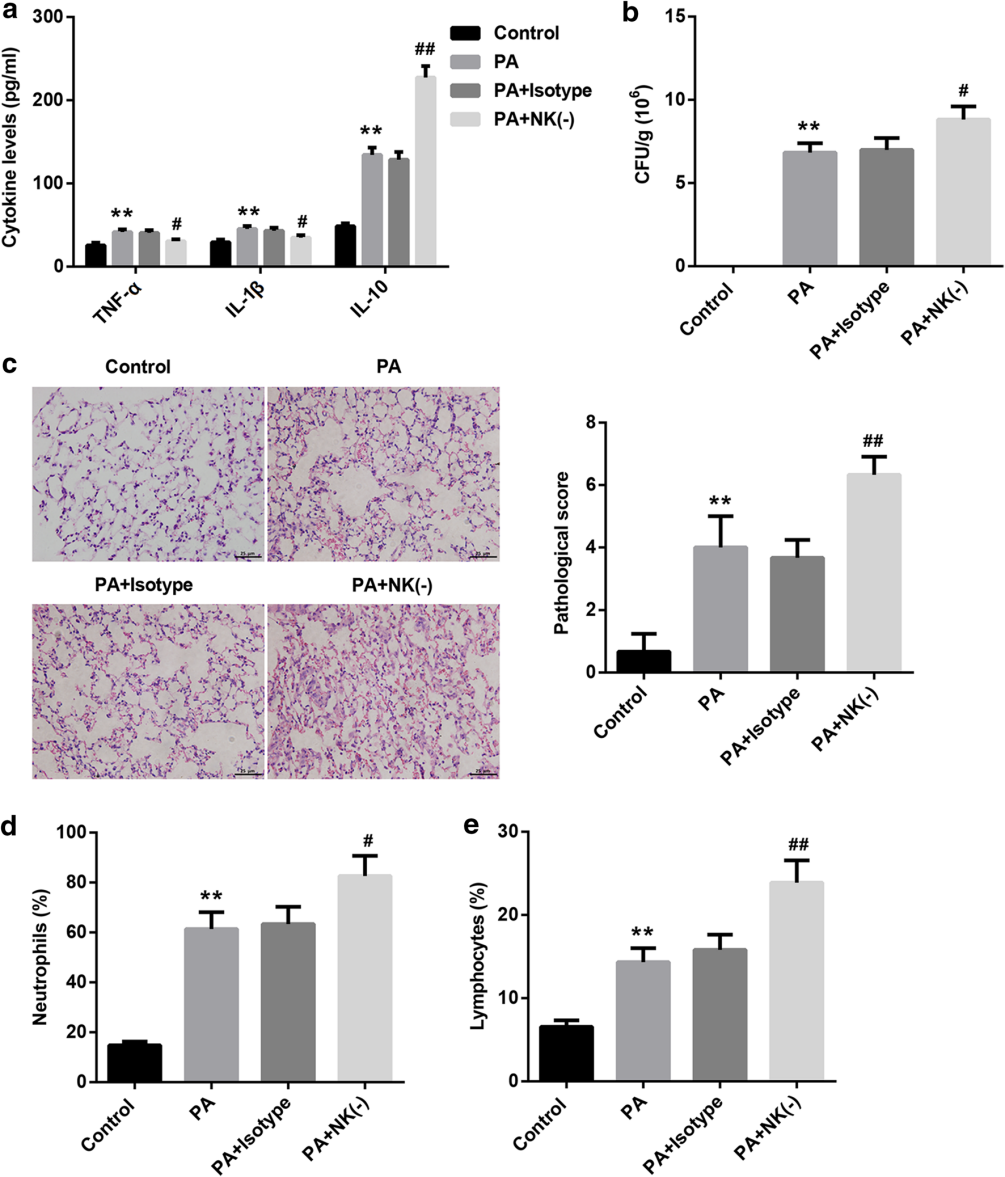
*PA*-induced lung injury was aggravated following NK cells elimination. **a** ELISA assay was performed to detect the concentration of TNF-α, IL-1β and IL-10 in lung tissues of each mouse. **b** The bacterial burden in the right lung tissues of each mouse was analyzed using CFU counting. **c** The pathological changes in lung tissues were confirmed by H&E staining. The scale bar is 25 μm. **d** The percentage of neutrophils in lung tissues from each mouse was ensured by flow cytometry. **e** Flow cytometry was carried out to examine the percentage of lymphocytes in lung tissues from each mouse. **P < 0.01 compared with Control group, both ^#^*P* < 0.05 and ^##^*P* < 0.01 contrasted with *PA* group

On the third day after surgery, the bacterial burden in right lung tissues of mice was confirmed by CFU counting. As shown in Fig. 1a, compared with control mice, the bacterial load in *PA*-infected mice was increased. Furthermore, to clear the effect of NK cells on macrophage polarization in *PA* lung infection, NK cell-depleted mice were induced by using anti-asialo GM1. Our data shown that the number of NK cells were significantly reduced in the lung tissues of NK cell-depleted *PA*-infected mice (Fig. 1b). Moreover, the number of CD86-positive cells (M1-polarized macrophage) was increased in *PA*-infected mice, while it was reduced after NK cell depletion. Besides, the number of CD206-positive cells (M2-polarized macrophage) was also increased in *PA*-infected mice. But, compared with CD86-positive cells, the percentage of CD206-positive cells was upregulated in *PA*-infected mice following NK cell depletion (Fig. 1c, d). Consistently, the markers of both M1-polarized macrophage, *iNOS*, and M2-polarized macrophage, *Arg-1*, were increased in the lung tissues of *PA*-infected mice. Interestingly, contrasted with *PA*-infected mice, the expression of *iNOS* in NK cell-depleted *PA*-infected mice was repressed, while *Arg-1* was boosted (Fig. 1e). The production of pro-inflammatory cytokines including TNF-α and IL-1β secreted by M1-polarized macrophage, and IL-10 from M2-polarized macrophage were facilitated by *PA* infection. Meantime, compared with *PA*-infected mice, the production of TNF-α and IL-1β was suppressed in NK cell-depleted *PA*-infected mice, while IL-10 production was promoted (Fig. 2a). The bacterial load in lung tissues of *PA*-infected mice was significantly increased, and NK cell-depleted mice with a higher bacterial load than non-depleted mice (Fig. 2b). The pathological changes in lung tissues were examined by using H&E staining. Our results indicated that *PA*-induced lung injury was notably aggravated following NK cell elimination (Fig. 2c). Besides, we also demonstrated that the percentages of neutrophil and lymphocyte in lung tissue of *PA*-infected mice were obviously higher than in the control mice, and the percentage of both neutrophils and lymphocytes was upregulated following NK cell deficiency (Fig. 2d, e). Overall, depletion of NK cells contributed to *PA*-induced lung injury via inducing M2 macrophage polarization; NK cells play a crucial role in *PA* lung infection.

### *NK cells boosted M1 macrophage polarization *via* secreting exosomes*

**Fig. 3:**
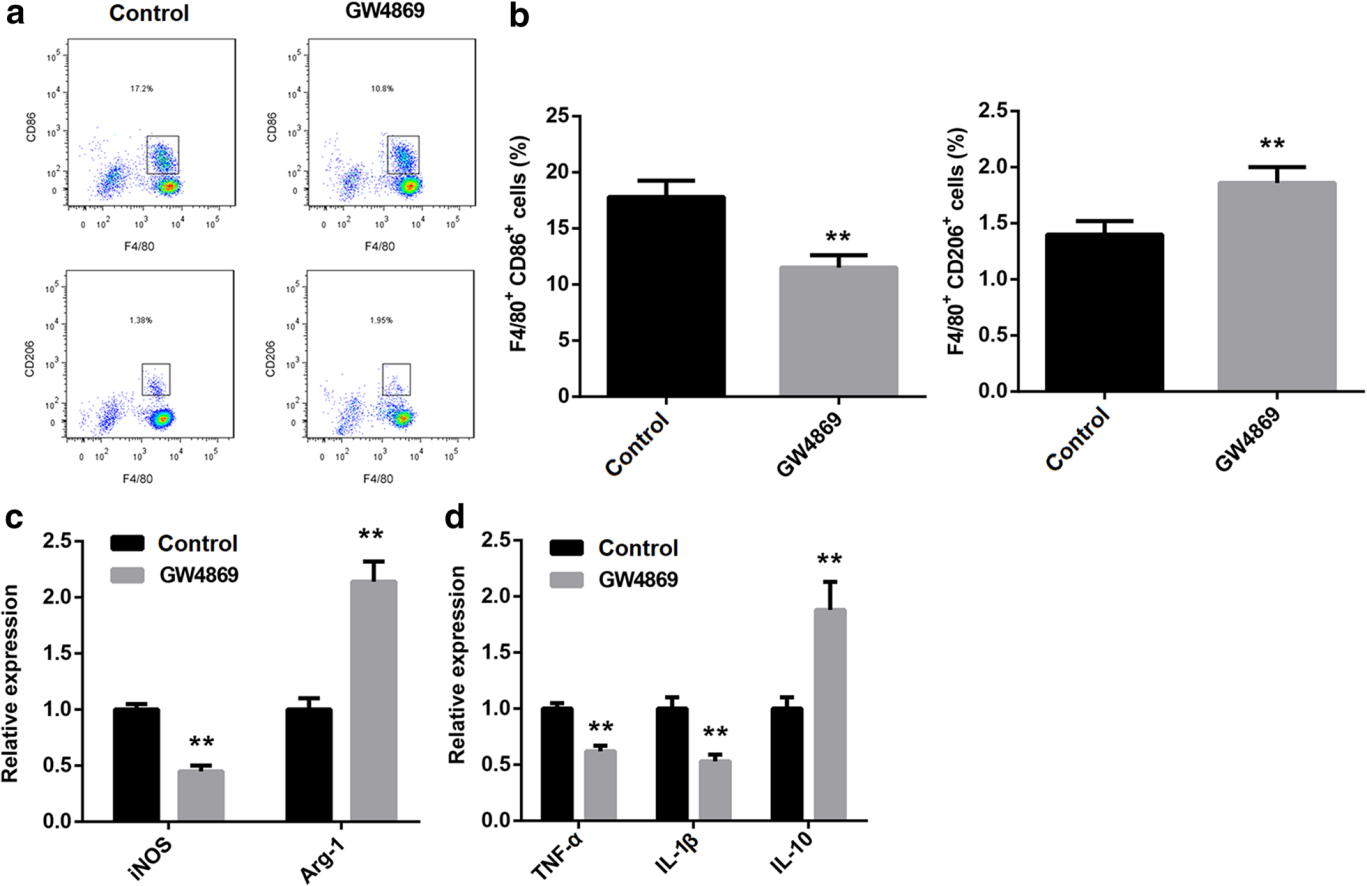
*PA*-infected macrophage to M2 polarization was facilitated following the ability of NK cells to secret exosomes was inhibited. The blocker of exosomes generation, GW4869, was used to stimulate NK cells, and then the cells were co-cultured with macrophage. **a** and **b** Flow cytometry was executed to examine the percentage of M1- and M2-polarized macrophage. **c** The relative expression of *iNOS* and *Arg-1* in macrophages was measured by qRT-PCR. **d** The expression of TNF-α, IL-1β and IL-10 in macrophages was detected by qRT-PCR. **P < 0.01 compared with Control group

**Fig. 4:**
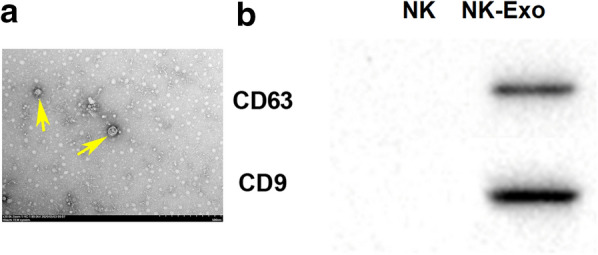
NK cell-derived exosomes detection. **a** The shape of exosomes was confirmed by transmission electron microscope. The yellow arrows were used to indicate NK cell-derived exosomes. Scale bar = 50 nm. **b** The expression of surface markers of exosome, including CD63 and CD9, was determined by western blot. NK-Exo: NK cell-derived exosome

**Fig. 5:**
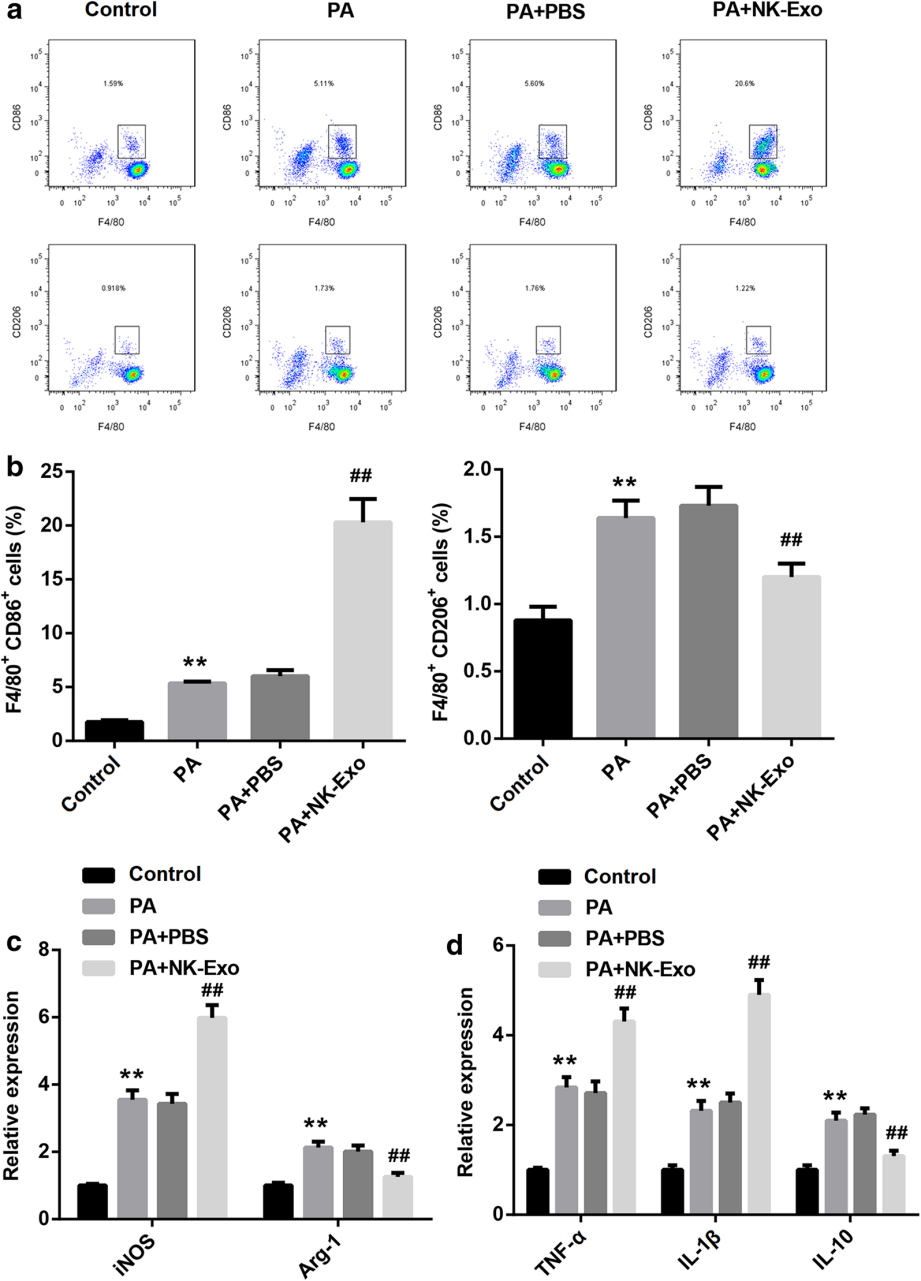
NK cells boosted *PA*-infected macrophage to M1 polarization through secreting exosomes. NK cell-derived exosomes or PBS were incubated with macrophage following *PA* infection. Then, **a** the percentage of M1- and M2-polarized macrophage were measured by flow cytometry. **b** The results of flow cytometry were analyzed and shown. **c** and **d** qRT-PCR was implemented to detect the expression of *iNOS* and *Arg-1* in macrophages, as well as the expression of TNF-α, IL-1β and IL-10. **P < 0.01 compared with Control group, and ^##^*P* < 0.01 contrasted with *PA* group

To explore the regulatory mode of NK cells in *PA* lung infection, we used GW4869, an inhibitor of exosomes, to stimulate NK cells which were then co-cultured with *PA*-infected macrophage. Our results proved that the number of M1-polarized macrophage was reduced in GW4869 group, while M2-polarized macrophage was increased (Fig. 3a, b). Besides, the expression of *iNOS* was downregulated in the macrophages co-cultured with GW4869-treated NK cells, while *Arg-1* expression was upregulated (Fig. 3c). Moreover, the expression of both TNF-α and IL-1β were downregulated in the macrophages which was co-cultured with GW4869-treated NK cells, but IL-10 expression was increased (Fig. 3d). The expression and secretion of IFN-γ were also reduced following the NK cells treated with GW4869 (Additional file 1: Figure S1a, b). In summary, the polarization of macrophage to M2 phenotype was facilitated when the generation of NK cell-derived exosome was blocked. Subsequently, NK cell-derived exosomes were collected and identified using transmission electron microscope (Fig. 4a). The expression of the surface markers of exosomes, including CD63 and CD9, was detected using western blot (Fig. 4b). To investigate whether NK cells regulate the polarization of macrophage through secreting exosomes, the macrophages were incubated with exosomes or PBS following *PA* infection, and then the polarization of macrophage was analyzed. The results of flow cytometry displayed that NK cell-derived exosomes could effectively promote M1 polarization and repress M2 polarization of *PA*-infected macrophages (Fig. 5a, b). QRT-PCR results demonstrated that the expression of *iNOS* was increased in the macrophages co-treated with *PA* and exosomes, and *Arg-1* expression was decreased (Fig. 5c). Besides, the expression of both TNF-α and IL-1β were promoted by NK cell-derived exosomes in *PA*-infected macrophage, while IL-10 expression was suppressed (Fig. 5d). In summary, our data indicated that NK cells promoted M1 polarization of *PA*-infected macrophage through secreting exosomes.

### *NK cells attenuated lung injury *via* secreting exosome in the mouse model of PA lung infection*

**Fig. 6:**
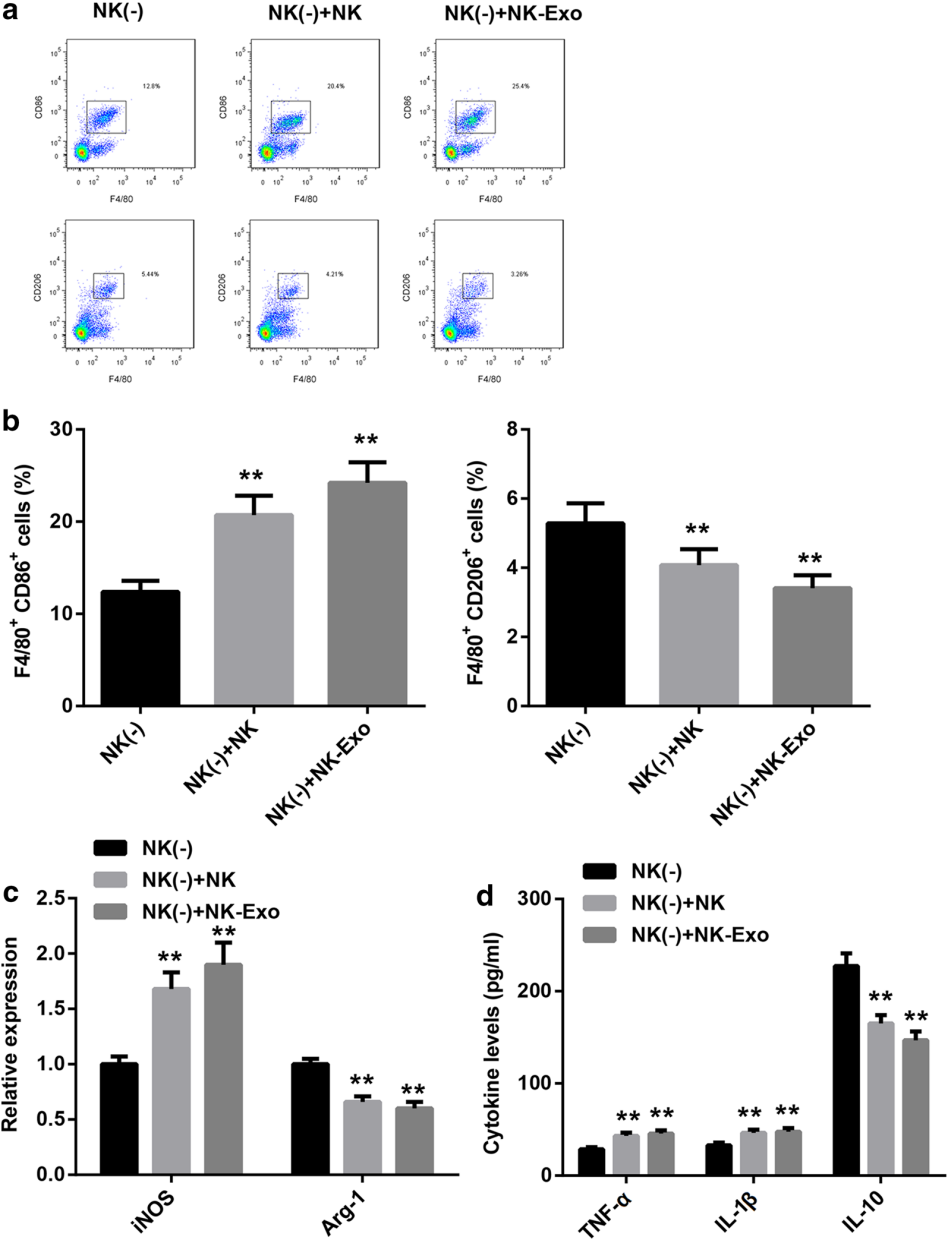
NK cells promoted macrophage M1 polarization through secreting exosomes. 1 × 10^7^ NK cells or 30 μg NK cell-derived exosomes were injected into the NK cell-depleted mice infected with *PA* in lung. Subsequently, **a** and **b** the percentage of M1- and M2-polarized macrophage in lung tissues was measured by flow cytometry, and then analyzed. **c** The expression of *iNOS* and *Arg-1* in lung tissues was ensured by qRT-PCR. **d** ELISA assay was carried out to determine the production of cytokines in lung tissues. **P < 0.01 compared with NK (-) group

**Fig. 7:**
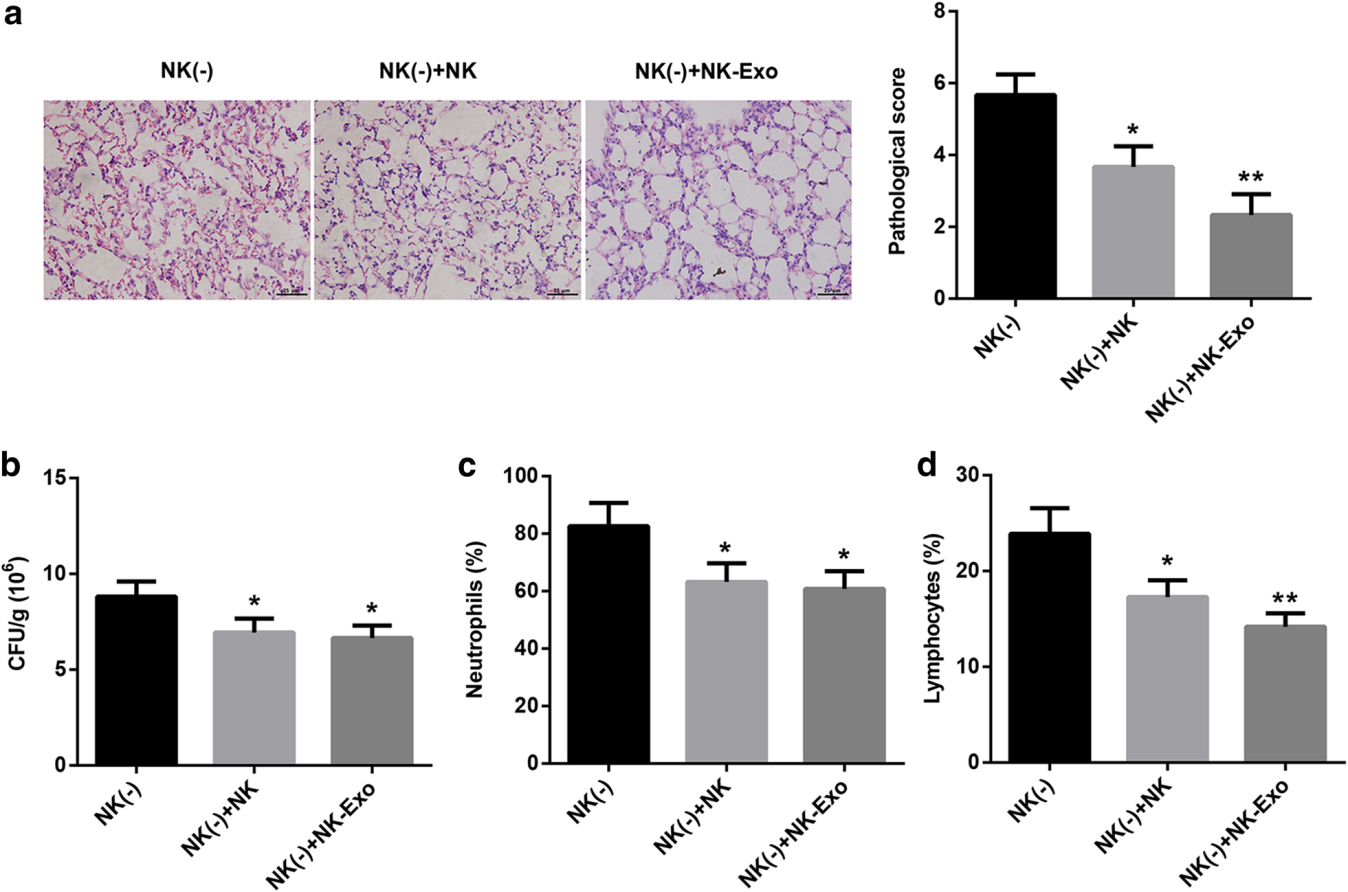
NK cells improved *PA*-induced lung injury through secreting exosomes. **a** H&E staining was used to analyze the pathological changes in lung tissues. The scale bar is 25 μm. **b** CFU counting was accomplished to examine the bacterial burden in the right lung tissues of each mouse. **c** and **d** The percentage of neutrophils and lymphocytes in lung tissues of each moues was measured by flow cytometry. *P < 0.05 and **P < 0.01 compared with NK (-) group

Next, we further clarified the action mechanism of NK cells in the mouse model of *PA* lung infection. NK cells or NK cell-derived exosomes were injected into NK cell-depleted mice following *PA* infection. At three days after surgery, the percentage of M1- and M2-polarized macrophage in lung tissues, and lung injury were analyzed. Here, our data indicated that both NK cells and its exosomes promoted macrophage to M1 polarization, and limited M2 polarization (Fig. 6a, b). Consistently, the expression of *iNOS* was increased by both NK cells and NK cell-derived exosomes, and *Arg-1* expression was repressed (Fig. 6c). ELISA assay results displayed that the production of TNF-α and IL-1β in lung tissues were boosted by both NK cells and NK cell-derived exosomes, while IL-10 production was impeded (Fig. 6d). Moreover, the improvement of NK cells and NK cell-derived exosomes to *PA*-induced lung injury was confirmed by H&E staining (Fig. 7a). The results of CFU counting indicated that both NK cells and NK cell-derived exosomes decreased the bacterial burden in lung tissues of NK cell-depleted mice (Fig. 7b). Furthermore, both NK cells and NK cell-derived exosomes reduced the percentage of neutrophils (Fig. 7c) and lymphocytes (Fig. 7d) in lung tissues of NK cell-depleted mice. Our data revealed that NK cells attenuated *PA*-induced lung injury of mice maybe via M1-polarized macrophage by secreting exosomes.

## Discussion

NK cells are the most crucial effector of innate lymphoid cells. NK cells were proved to mediate and/or amplify inflammatory response, and act as a major regulator in defense against bacterial and viruses, as well as tumor immune-surveillance [22]. Normally, activated NK cells will infiltrate into the bacterial-infected tissues to against pathogen invasion. However, the cytotoxicity of NK cells may exacerbate the development of immune disease under certain pathological conditions [23, 24]. It was indicated that in the mouse model of *PA* ocular infection, the percentage of NK cell in corneal tissues is increased with the disease development [25]. In addition, Svetlana et al. displayed that the number of NK cell in lung tissues of wild-type mouse is also increased following *PA* lung infection [26]. Increased NK cell in lung tissues of *PA*-infected mouse model was also found by Scott C et al. [27]. Overall, NK cells play a crucial role in the development of *PA* lung infection. Here, we used an antibody of anti-asialo GM1 to clear the NK cells in mouse *PA* lung infection model. Then, we found that elimination of NK cells aggravated *PA*-induced lung injury.

Wu et al. demonstrated that NK cell depletion could improve paraquat dichloride-induced lung injury in mouse via reversing macrophage M1 polarization [28]. Tissue-resident macrophages are present in almost all the organs of human, the reversibility of its polarization has a great therapeutic value in multiple diseases, especially in the diseases with an imbalanced M1/M2 macrophage percentage [29]. During the healing of a healthy injury without infection, macrophage will pass the message of inflammation resolution following the apoptotic neutrophils were devoured. However, upon bacterial infection such as *PA* infection, the process of normal inflammation subsides and healing will be interrupted [30]. At present, there have been limited research to explore the interaction between *PA* and macrophage. It was proved that the macrophages are recruited in the lung tissues, and the production of pro-inflammatory factors (TNF-α, IL-6, IL-1β and IL-1α) are increased in the lung tissues and bronchoalveolar lavage fluid of mice following *PA* lung infection [31]. It is necessary to explore the relationship between *PA* lung infection and macrophages. Small et al. revealed that lung macrophages can better wipe out *Staphylococcus aureus* under the condition of NK cells presence in the co-culture system of macrophage and NK cells [32]. Nicolas et al. indicated that after *salmonella* infection, activated NK cells could significantly reduce the number of bacteria in macrophage, and the crosstalk between infected macrophages and NK cells is contact-dependent [33]. The crosstalk between macrophages and NK cells is fascinating and has drawn widespread attention, especially in the study of bacterial infection [34]. In the present study, our results demonstrated that the number of macrophage in lung tissues of mice was increased following *PA* infection, while NK cell depletion leaded to M2 lung macrophage polarization in the mouse model of *PA* lung infection.

Exosomes are nanovesicles with a diameter of 40–100 nm. Exosomes can be released by almost all cell types, such as macrophage and NK cell. Exosomes act as an important megaphone between cells and cells [35]. It was demonstrated that exosomes play a crucial role in multiple pathological and physiological processes, including immune responses, neural communication, reproduction and development, tumor pathogenesis, bacterial and viral infection [36, 37]. NK cells are majorly involved in the killing of virus and bacterial infection and tumor cells, during the process of immune response. NK cells can communicate with other immune cells through exosomes [14]. Here, we found firstly that inhibition of the generation of exosomes from NK cells led to the M2 polarization of macrophage. Then, we further indicated that NK cells promoted M1 polarization of macrophage infected with *PA*, and attenuated *PA*-induced lung injury via secreting exosomes.

## Conclusion

Overall, our data indicated that NK cells promoted M1 macrophage polarization and attenuated lung injury by secreting exosomes in the mouse model of *PA* lung infection. Our data may provide a new idea for the treatment of *PA* lung infection. However, there are yet many works that need to be done. Generally, the polarization toward anti-inflammatory M2 macrophage reduces the inflammation and subsequently improves the injury. But, in our study, we indicated that NK cells promoted macrophage M1 polarization to attenuate lung injury. We conjecture that M1 polarization promotes the phagocytosis of macrophage in the early stage of lung *PA* infection. Hence, more molecular mechanisms will be explored in our following study.

### Electronic supplementary material

Below is the link to the electronic supplementary material.

**Additional file 1: Figure S1.** The expression of IFN-γ in NK cells treated with GW4869.

## Data Availability

The datasets used and/or analyzed during the current study are available from the corresponding author on reasonable request.
