# Supplementary figures and images for: NK cell-derived exosomes improved lung injury in mouse model of Pseudomonas aeruginosa lung infection
Source: J Physiol Sci. 2020 Oct 23;70:50. doi: 10.1186/s12576-020-00776-9 (PMC10717361; doi:10.1186/s12576-020-00776-9)

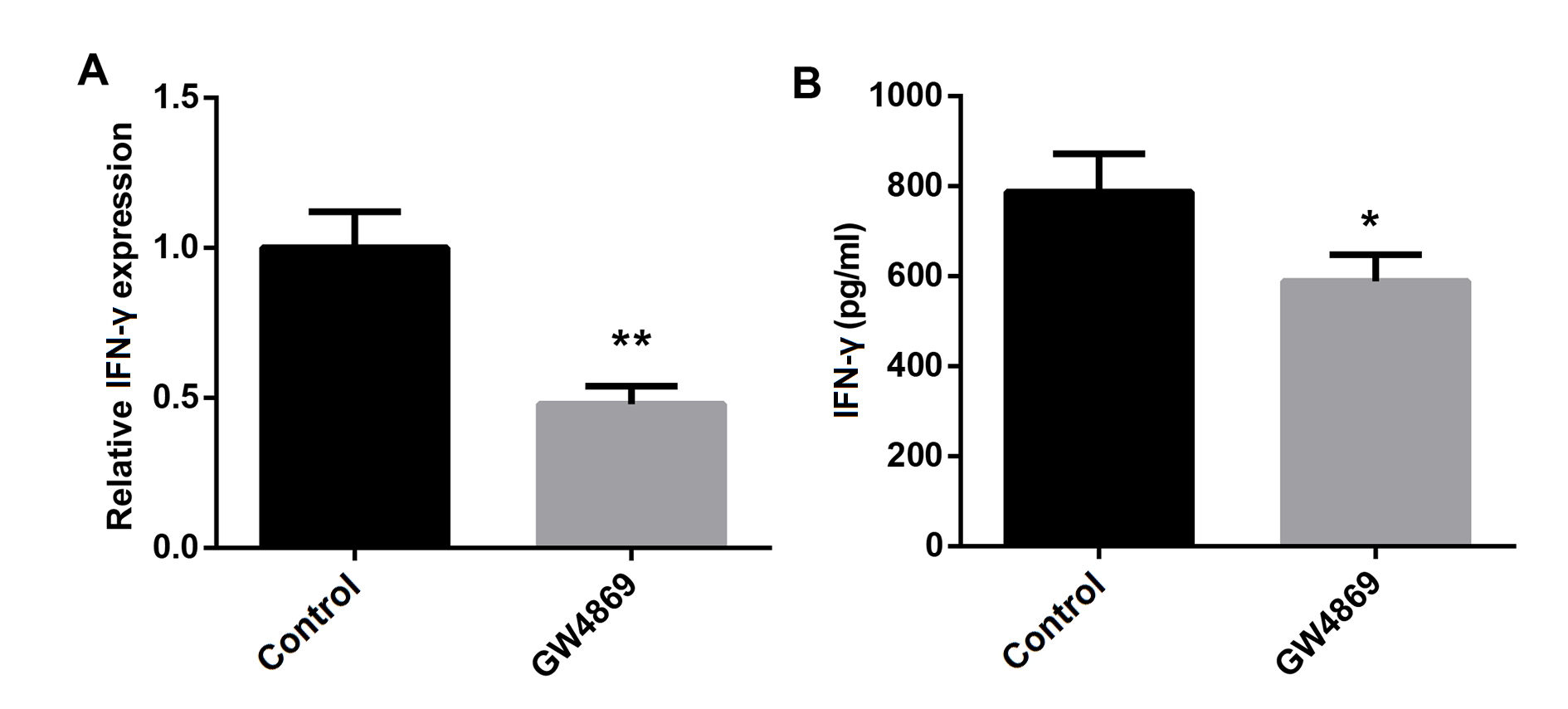

Supplement: Supplementary file 1 — Additional file 1: Figure S1. The expression of IFN-γ in NK cells treated with GW4869. [file 12576_2020_776_MOESM1_ESM.tif]
